# Extract from *Aronia melanocarpa*, *Lonicera caerulea*, and *Vaccinium myrtillus* Improves near Visual Acuity in People with Presbyopia

**DOI:** 10.3390/nu16070926

**Published:** 2024-03-23

**Authors:** Dorota Szumny, Alicja Zofia Kucharska, Karolina Czajor, Karolina Bernacka, Sabina Ziółkowska, Patrycja Krzyżanowska-Berkowska, Jan Magdalan, Marta Misiuk-Hojło, Tomasz Sozański, Adam Szeląg

**Affiliations:** 1 Department of Pharmacology, Wroclaw Medical University, Mikulicza-Radeckiego 2, 50-345 Wroclaw, Poland; jan.magdalan@umw.edu.pl (J.M.); adam.szelag@umw.edu.pl (A.S.); 2 Ophthalmology Clinic, University Clinical Hospital, Borowska 213, 50-556 Wrocław, Poland; karolina.czajor@gmail.com (K.C.); sabinaziolkowska@gmail.com (S.Z.); p_krzyzanowska@wp.pl (P.K.-B.); misiuk55@wp.pl (M.M.-H.); 3 Department of Fruit, Vegetable and Plant Nutraceutical Technology, Wrocław University of Environmental and Life Sciences, Chełmońskiego 37, 51-630 Wrocław, Poland; alicja.kucharska@upwr.edu.pl (A.Z.K.); karolina.bernacka@upwr.edu.pl (K.B.); 4 Department of Ophthalmology, Wroclaw Medical University, Borowska 213, 50-556 Wrocław, Poland; 5 Department of Preclinical Sciences, Pharmacology and Medical Diagnostics, Faculty of Medicine, Wroclaw University of Science and Technology, Wybrzeże Wyspiańskiego 27, 50-370 Wrocław, Poland

**Keywords:** anthocyanins, dry eye syndrome, iridoids, natural extracts, presbyopia

## Abstract

Presbyopia is a global problem with an estimated 1.3 billion patients worldwide. In the area of functional food applications, dietary supplements or herbs, there are very few reports describing the positive effects of their use. In the available literature, there is a lack of studies in humans as well as on an animal model of extracts containing, simultaneously, compounds from the polyphenol group (in particular, anthocyanins) and iridoids, so we undertook a study of the effects of a preparation composed of these compounds on a condition of the organ of vision. Our previous experience on a rabbit model proved the positive effect of taking an oral extract of *Cornus mas* in stabilizing the intraocular pressure of the eye. The purpose of this study was to evaluate the effect of an orally administered ternary compound preparation on the status of physiological parameters of the ocular organ. The preparation contained an extract of the chokeberry *Aronia melanocarpa,* the honeysuckle berry *Lonicera caerulea* L., and the bilberry *Vaccinium myrtillus* (hereafter AKB) standardized for anthocyanins and iridoids, as bioactive compounds known from the literature. A randomized, double-blind, cross-over study lasting with a “wash-out” period of 17 weeks evaluated a group of 23 people over the age of 50, who were subjects with presbyopia and burdened by prolonged work in front of screen monitors. The group of volunteers was recruited from people who perform white-collar jobs on a daily basis. The effects of the test substances contained in the preparation on visual acuity for distance and near, sense of contrast for distance and near, intraocular pressure, and conjunctival lubrication, tested by Schirmer test, LIPCOF index and TBUT test, and visual field test were evaluated. Anthocyanins (including cyanidin 3-O-galactoside, delphinidin 3-O-arabinoside, cyanidin 3-O-glucoside, cyanidin 3-O-rutinoside, cyanidin 3-O-arabinoside) and iridoids (including loganin, sweroside, loganic acid) were identified as substances present in the extract obtained by HPLC-MS. The preliminary results showed that the composition of AKB applied orally does not change visual acuity in the first 6 weeks of administration. Only in the next cycle of the study was an improvement in near visual acuity observed in 92.3% of the patients. This may indicate potential to correct near vision in presbyopic patients. On the other hand, an improvement in conjunctival wetting was observed in the Schirmer test at the beginning of week 6 of administration in 80% of patients. This effect was weakened in subsequent weeks of conducting the experiment to 61.5%. The improvement in conjunctival hydration in the Schirmer test shows the potential beneficial effect of the AKB formulation in a group of patients with dry eye syndrome. This is the first study of a preparation based on natural, standardized extracts of chokeberry, honeysuckle berry, and bilberry. Preliminary studies show an improvement in near visual acuity and conjunctival hydration on the Schirmer test, but this needs to be confirmed in further studies.

## 1. Introduction

Presbyopia is a progressive loss of accommodation, resulting in a loss of visual ability to focus on objects at different distances. Presbyopia is a global problem, estimated at 1.3 billion patients worldwide and on an ever-increasing trend [1,2].

It is now thought to be a consequence of two reasons: first, the design of the transparent lens and the way it must change shape to allow focusing, and second, the instability of the lens proteins over very long periods of time [3]. Loss of lens elasticity may be related to an increase in disulfide bond formation in the collagen of the aging lens, possibly due to oxidative stress. Increasing changes in the lens lead to a loss of elasticity in middle age, with a consequent onset of presbyopia, and may probably also promote the later development of cataracts [4].

In humans, accommodation occurs through contraction of the ciliary muscle and iris sphincter and convergence and changes in the shape and position of the lens. Stimulation of the parasympathetic nervous system affects accommodative abilities [5]. 

To date, presbyopia is standardly corrected with glasses, as well as with contact lenses and surgery [5,6,7]. 

In recent years, two types of pharmacological effects on presbyopia have been studied: pupil-constricting agents and agents that improve lens elasticity (e.g., 1.5% lipoic acid choline ester solution) [5,8]. Pupil-constricting agents, such as 1.25% pilocarpine, stimulate accommodation by creating a pinhole effect that can increase depth of field at all distances and stimulating muscarinic receptors [8]. 

Preparations of natural origin in the form of dietary supplements or herbs are used in the adjunctive treatment of eye disorders, both for external application in conjunctival disorders and for oral use, such as dietary supplements recommended for the dry form of age-related macular degeneration. Examples of plant compounds belonging to the carotenoids that have found wide use in ophthalmology for the treatment of age-related macular degeneration are the tertaterpenoids lutein and zeaxanthin extracted from plant material. Their oral administration slows macular degeneration in about 25% of patients compared to a control group (AREDS study) [9,10].

One of the first plants widely used to improve vision was bilberry, whose oral administration caused patients to have an increased sense of contrast and improved night vision and had antioxidant effects [10].

Substances of natural origin such as iridoids and anthocyanins have been studied as potentially useful in the treatment of eye diseases [11].

Iridoids are secondary metabolites present in a variety of plants, especially in species belonging to the Apocynaceae, Lamiaceae, Loganiaceae, Rubiaceae, Scrophulariaceae, and Verbenaceae families. Many of these substances have a long history of traditional use in natural medicine. Iridoids exhibit a wide range of pharmacological activities, such as cardiovascular, hepatoprotective, hypoglycemic, antimutagenic, antispasmodic, anticancer, antiviral, immunomodulatory, and purgative effects, as well as anti-inflammatory effects [12,13]. In traditional medicine, extracts or infusions of eyebright (Euphrasia rostkoviana), containing the iridoid, aucubin, were used for dry eye syndrome and other eye diseases [14,15,16].

Our previous experience of using monoterpenoid sugar derivatives with the iridoid system—loganic acid, the active ingredient of *Cornus mas*—has shown their effectiveness in lowering intraocular pressure on a rabbit model after oral administration [17]. 

Anthocyanins are powerful antioxidants that scavenge radicals and chelate metal ions. Many herbs and berries have strong antioxidant properties, and these properties are believed to underlie many of the various health effects of these raw materials [10]. Administration of anthocyanins has a relaxing effect on ciliary muscles, which is important in the treatment of both myopia and glaucoma. Anthocyanins improved dark adaptation and a transient shift toward myopia and improved retinal blood circulation in glaucoma patients with normal pressure [11]. The antioxidant effects of anthocyanins contained in edible berry extracts have also been confirmed [11]. A chokeberry extract shows antioxidant activity and anti-inflammatory effect in induced uveitis in rats [18]. Orally administered anthocyanoside oligomers in myopic patients with asthenopia resulted in improved visual acuity and sense of contrast [19]. Natural compounds such as polysaccharides and betaine contained in Goya berries caused a reduction in dry eye syndrome symptoms in an experimental study in rats [20].

A frequently observed ophthalmic problem that patients face in addition to presbyopia is dry eye syndrome. It is a multifactorial disorder characterized by loss of tear film homeostasis with an estimated prevalence of 5% to 50% worldwide. The standard treatment is eye drops containing moisturizers such as hyaluronic acid or lubricants that reduce tear evaporation. Various substances are being researched for administration into the conjunctival sac as well as orally, and this group also includes compounds of plant origin [21]. Although dietary supplements to alleviate the symptoms of dry eye containing plant extracts, e.g., *Aristotelia chilensis*, are known on the pharmaceutical market, their effects of improving near vision are unknown. 

The available literature lacks human studies of standardized extracts containing simultaneously polyphenol group compounds (especially anthocyanins) and iridoids. The authors found only a few papers describing the effectiveness of orally administered herbal extracts in the prevention and supporting pharmacological treatment of presbyopia. No literature reports have described the human activity of the preparation described in this paper. The positive effect of wild blueberry extracts on near vision and mucosal hydration is known. However, the limited availability of wild-caught blueberries as well as their highly variable composition (depending on harvest region, climate, soil conditions, etc.) is a real problem for the production of a dietary supplement. In view of the above, work on finding effective, easily cultivated substitutes with proven effects, i.e., chokeberry, honeysuckle berry, seems to be highly advisable. Work on finding substitutes for bilberry fruit was carried out by Ogawa et al., where the authors investigated the possibility of using the more accessible leaves of this plant [22].

Therefore, this study was undertaken to evaluate a preparation composed of chokeberry, honeysuckle berry (Kamchatka berry), and bilberry edible fruit extracts containing anthocyanins and iridoids, marketed as a dietary supplement, on ocular conditions. The intervention dietary study evaluated a group of people burdened with long-term work in front of screen monitors and with developing presbyopia. The effects of the test substances on distance and near visual acuity, sense of contrast, conjunctival hydration, and visual field were evaluated. Approval from the Local Bioethics Committee No. KB-672/2022 was obtained for the study.

## 2. Materials and Methods

### 2.1. Chemicals

Acetonitrile and 98–100% formic acid were used from Merc (Darmstadt, Germany). The standards of phenolic compounds and iridoids were purchased from Extrasynthese (Genay, France). All reagents were of analytical or HPLC. 

### 2.2. Preparation of AKB Extract

Extracts were manufactured at industrial scale by Greenvit, Zambrów (Poland)—botanical extracts manufacturer. Preparation starts from frozen berries, using acidified water as solvent (lower pH stabilize anthocyanins); after extraction by percolation, the liquid extract is separated from exhausted biomass, filtered, and then purified on an adsorption resin bed. After the resin column is saturated, the concentrated extract is eluted by water–ethanol solution, then water–ethanol is evaporated, and finally, the extract is spray-dried, with some addition of carrier (maltodextrin) to standardize the active ingredients (anthocyanins) to min. 25% by HPLC. At the end, 3 standardized dry extracts are blended in an industrial blender (ribbon mixer) in a 2:1:1 ratio to get a homogenous blend called Oculoxin^®^. The final blend is again analyzed, and if the batch meets the specification, it is released by the Quality Department, and CoA is issued. Before the study, the final blend is then encapsulated into hard gel capsules, using a semi-automatic capsule-filling equipment. 

### 2.3. Quantification of Compounds by HPLC-PDA

The quantification of compounds was performed according to the method previously described by Przybylska et al. [23], using a Dionex (Germering, Germany) system, equipped with the diode array detector model Ultimate 3000, quaternary pump LPG-3400A, autosampler EWPS-3000SI, thermostated column compartment TCC-3000SD, and controlled by Chromeleon 7.2 software (Thermo Scientific Dionex, Sunnyvale, CA, USA). Cadenza Imtakt column CD-C18 (75 × 4.6 mm, 5 μm) with a guard column at 30 °C. The following eluents were used: A, 4.5% aq. formic acid, *v*/*v* and B, 100% acetonitrile, and the gradient profile was as follows: 0–1 min 5% B in C, 1–20 min 25% B in C, 20–21 min 100% B, 21–26 min 100% B, and 26–30 min 5% B in A. The flow rate of the mobile stage was 1 mL/min, and the injection volume was 20 μL. The UV–Vis measurements were made in the wavelength range of 200–600 nm in steps of 2 nm. Iridoids were detected at 245 nm, phenolic acids at 320 nm, flavonols at 360 nm, and anthocyanins at 520 nm, and these were quantified using linear regression equations based on external standards. Results are provided as the mean ± standard deviation of three replications and expressed as mg/100 g of dry weight (dw) of extract.

### 2.4. Composition of the Preparation (Composition of Extracts) AKB Prepared for the Study

**Figure 1:**
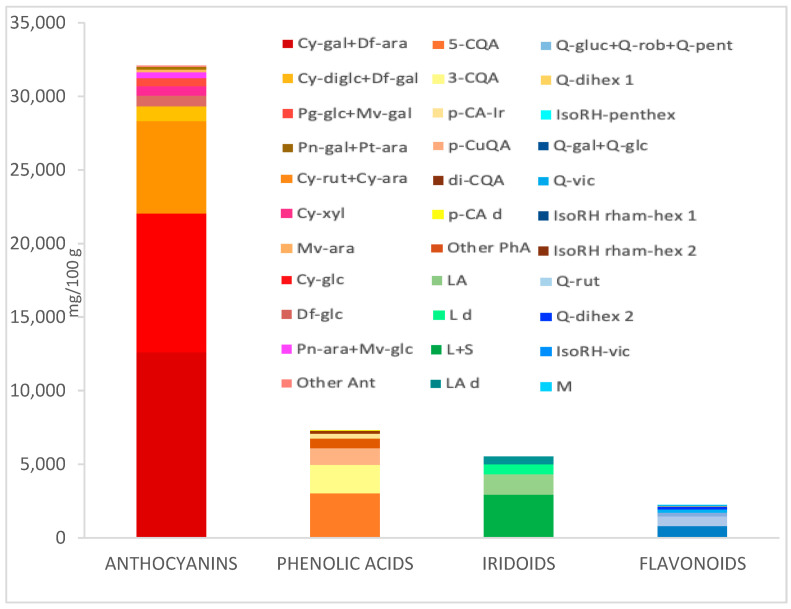
The content (mg/100 g dry mass) of phenolic and iridoid compounds of the extract (AKB) by HPLC-PDA. Abbreviations: Cy-gal-Cyanidin 3-O-galactoside; Df-ara-Delphinidin 3-O-arabinoside; Cy-glc-Cyanidin 3-O-glucoside; Cy-rut-Cyanidin 3-O-rutinoside; Cy-ara-Cyanidin 3-O-arabinoside; Cy-diglc-Cyanidin 3,5-O-diglucoside; Df-gal-Delphinidin 3-O-galactoside; Df-glc-Delphinidin 3-O-glucoside; Cy-xyl-Cyanidin 3-O-xyloside; Pg-glc-Pelargonidin 3-O-glucoside; Mv-gal-Malvidin 3-O-galactoside; Pn-ara-Peonidin 3-O-arabinoside; Mv-glc-Malvidin 3-O-glucoside; Mv-ara-Malvidin 3-O-arabinoside; Pn-gal-Peonidin 3-O-galactoside; Pt-ara-Petunidin 3-O-arabinoside; Ant-Anthocyanins; 5-CQA-5-Caffeoylquinic acid; 3CQA-3-Caffeoylquinic acid; *p*-CuQA-3-O-p-coumaroylquinic acid; PhA-phenolic acids; *p*-CA-Ir-Coumaroyl iridoid glycosides; di-CQA-Dicaffeoylquinic acid; *p*-CA d-p-Coumaric acid derivative; L-Loganin; S-Sweroside; LA-loganic acid; d-derivative; Q-gal-Quercetin 3-O-galctoside; Q-glc-Quercetin 3-O-glucoside; Q-rut-Quercetin 3-O-rutinoside; Q-gluc-Quercetin 3-O-glucuronide; Q-rob-Quercetin 3-O-robinobioside; Q-pent-Quercetin 3-O-pentoside; Q-vic-Quercetin 3-O-vicianoside; Q-dihex 1-Quercetin-dihexoside isomer 1; Q-dihex 2-Quercetin-dihexoside isomer 2; IsoRH rham-hex 1-Isorhamnetin rhamnosylhexoside isomer 1; IsoRH-vic-Isorhamnetin 3-O-vicianoside; IsoRH-pent-hex-Isorhamnetin pentosylhexoside; IsoRH rham-hex 2-Isorhamnetin rhamnosylhexoside isomer 2; M-Myricetin.

**Figure 2:**
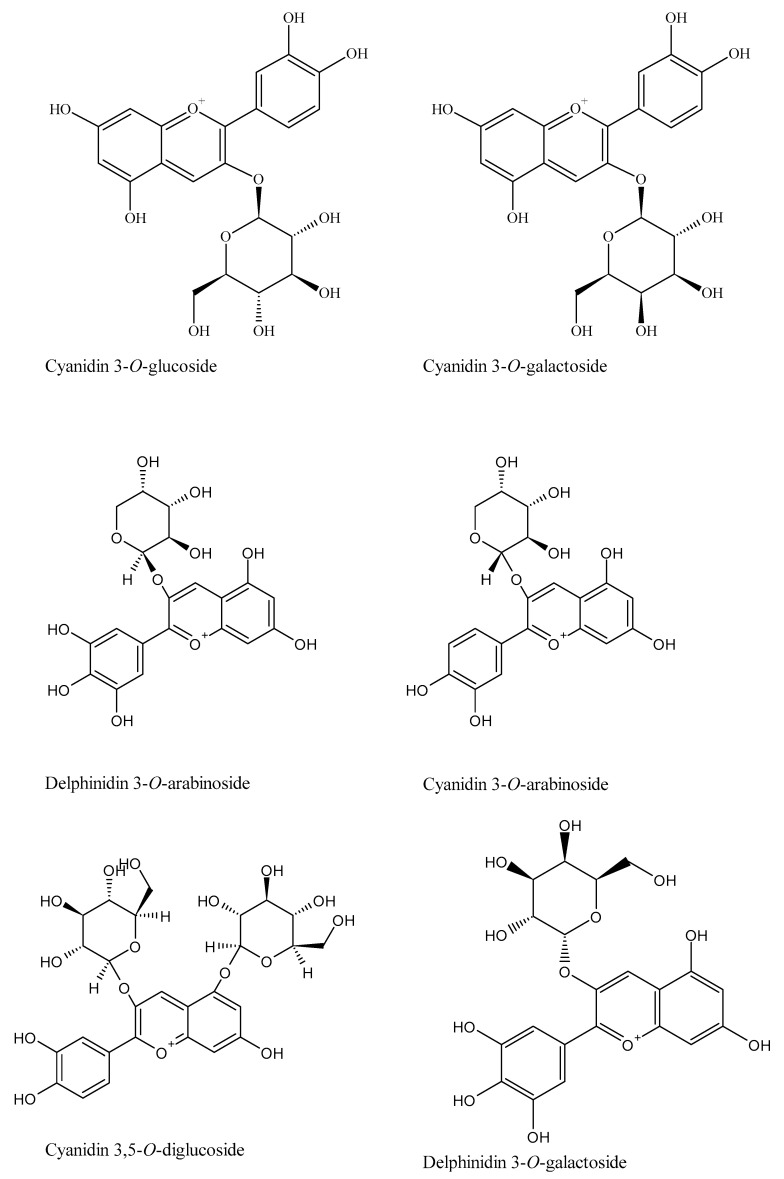

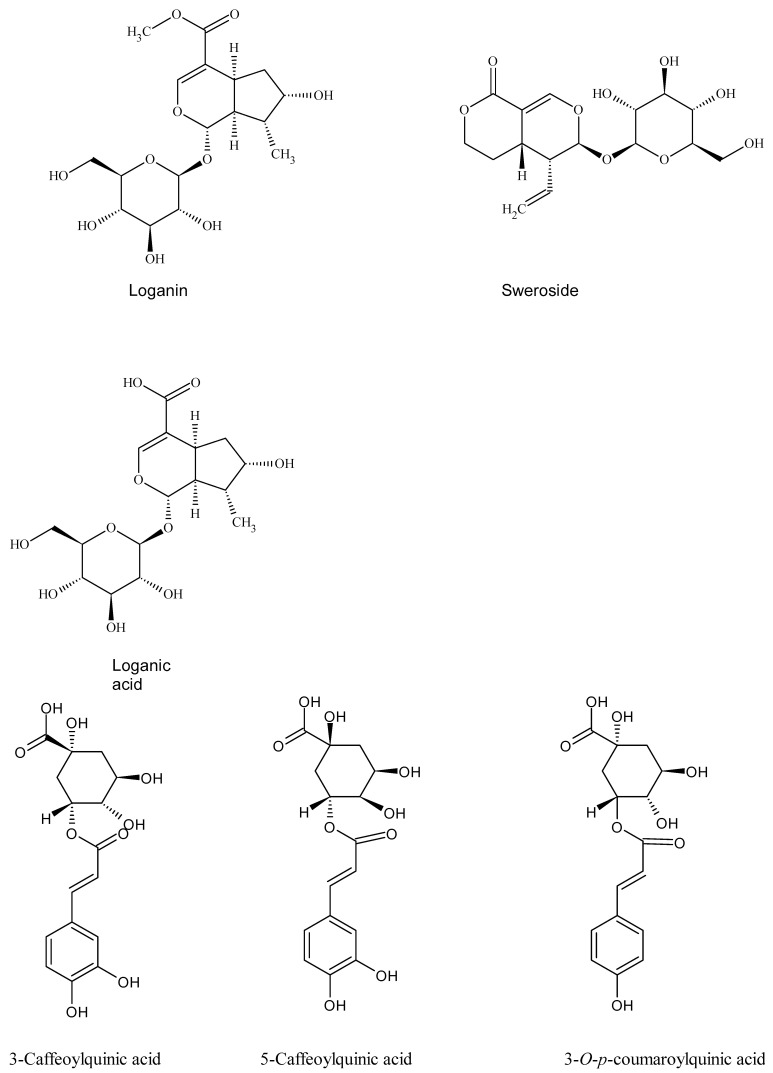
Chemical structures of main compounds from each group found in AKB extract.

The orally administered AKB dietary supplement, doubly standardized for anthocyanins and iridoids, is a blend of aronia/chokeberry (A), kamchatka/honeysuckle berry(K), and bilberry (B) fruit extracts in a 2:1:1 ratio. The quantitative composition of purified fruit extracts per AKB capsule was as follows: 200 mg of dry extract from chokeberry fruit, 100 mg of dry extract from honeysuckle berry fruit, 100 mg of dry extract from bilberry fruit. The standardized content of anthocyanins was no less than 25% and iridoids, no less than 4.5%. A mixture of AKB plant extracts was administered, daily dose: two capsules each of 400 mg twice a day. Figure 1 shows the detailed composition of the preparation. The results are given in mg per 100 g of powder. Anthocyanins (32,102.09 mg/100 g) predominated in the extract studied, followed by phenolic acids (7322.09 mg/100 g), iridoids (5529.41 mg/100 g), and flavonols (2240.48 mg/100 g). Among the anthocyanins, 16 compounds were identified, i.e., most of the red pigments included in bilberry, chokeberry, and kamchatka berry. Phenolic acids were mainly 3 cavoylquinone derivatives (5-CQA > 3-CQA > di-CQA) and 3 *p*-coumaric acid derivatives, including coumaroyl iridoid glycosides from bilberry. The source of simple iridoids including secoiridoids was Kamchatka berry. In the AKB preparation, 5 compounds from this group were identified, i.e., loganin, sweroside, loganic acid, and their derivatives. A total of 14 flavonols were determined, among which quercetin derivatives predominated, followed by isoramnetin. Chemical structures of main compounds presented in extract are shown in Figure 2 and supplementary date (Table S1).

### 2.5. Study Group, Conditions for Inclusion in the Study

The study included 30 volunteers. Inclusion criteria for patients were as follows: age over 50 with presbyopia best corrected visual acuity ≥ 20/40, spherical refraction of −3 to +3 diopters, and cylinder correction within ±3.0 diopters. Subjects were excluded if they had a history of ocular surgery within 12 months before the onset of the study. Patients younger than 50 years old, with intraocular disease (e.g., macular degeneration, diabetic retinopathy, retinal vein occlusion, glaucoma, significant acquired or hereditary eye diseases) or neurological disorders affecting visual fields were also excluded from the study. Finally, patients with hypotension, severe circulatory failure, or other vascular endothelial abnormalities that could impact the optic nerve head blood flow and patients with diseases of the gastrointestinal tract (such as hyperacidity, peptic ulcer disease) were also excluded from the study.

In the qualifying medical examination (history), 3 people were eliminated due to existing exclusionary conditions. Subsequently, after initiating diagnostic testing but before administering the products, 2 people were found to have the initial stage of glaucoma, and 1 person was found to have a visual impairment caused by a general disease. One person dropped out of further study after the first stage. 

The full cycle of the study involved 23 people, divided into two groups of 10 and 13 people, respectively. 

### 2.6. The Study Being Conducted 

The study was conducted at the Department of Ophthalmology of the University Clinical Hospital in Wroclaw, based on an approved application to the Bioethics Committee at the Medical University of Wroclaw (No. KB-672/2022). The study was implemented according to the Principles of Good Clinical Practice (GCP).

The study was planned and executed as a randomized, double-blind, cross-over study. It was divided into two, separated by a pause (wash-out) period (Stage 1 and Stage 2). Each stage involved testing the same parameters before and after participants completed use of the assigned product. The results obtained from the 2 stages of the study were subject to further analysis.

The duration of Stage 1 of the study was 6 weeks (42 days). It was followed by a “wash-out” period lasting 5 weeks. Subsequently, stage 2 of the study also lasted 6 weeks (42 days). In each stage, patients alternately took one capsule of the dietary supplement or a placebo twice a day. The product capsule contained standardized chokeberry, kamchatka/honeysuckle berry, and bilberry fruit extract (AKB) in a ratio of 2:1:1. Patients receiving the placebo received one capsule twice a day containing the natural dye caramel, suspended in maltodextrin.

A cross-over study is a research design that allows the comparison of two interventions (in this case, investigational product–placebo) in the same set of participants. In a cross-over study, each participant is alternately given the investigational product and placebo at different times in random order. This allows the efficacy and safety of the therapy to be compared in the same group of people, eliminating the influence of inter-individual variables. 

Patients were examined ophthalmologically before starting the dietary supplement and during the last week of taking it. The following ophthalmologic examinations were performed: visual acuity for distance and near, intraocular pressure, contrast sense test for distance and near, visual field, tests for dry eye syndrome (Schirmer test, LIPCOF test, tear film break time tBUT).

In the case of the nearsightedness test and the Schirmer test, the researchers’ presumed clinical benefit was an improvement in any eye of the subject.

Statistical analyses were carried out using the IBM SPSS Statistics 26 package, using which a chi-square test of concordance and a chi-square test of independence were performed. The significance level in this paper was considered to be α = 0.05.

## 3. Results

Among the pre-tested samples, chokeberry extract (A) is the richest source of anthocyanins and phenolic acids, kamchatka berry extract (K) is exceptionally rich in simple iridoids, which are not found in chokeberry and bilberry, while bilberry extract (B) has an unusually rich qualitative composition of anthocyanins (five different aglycones linked in monoglycoside form with hexose or pentose).

The qualitative composition of the active compounds of chokeberry, kamchatka berry, and bilberry fruits is well known and described. Chokeberry fruits are a rich source of polyphenolic compounds, among which anthocyanins (mainly cyanidin-3-galactoside, followed by cyanidin-3-arabinoside and, in much lower amounts, cyanidin-3-glucoside and cyanidin-3-xyloside), procyanidins to a high degree in polymerized form, and phenolic acids (mainly, cavoylquinic acids) predominate [24,25]. They also contain carotenes like *β*-carotene and xanthophylls like lutein and *β*-cryptoxanthin [26].

Honeysuckle berry fruits contain polyphenols, iridoids, and, in smaller amounts, carotenoids, among others. Kucharska at al. (2017) identified 50 compounds [24]. Among the polyphenols were six anthocyanins (mainly cyanidin 3-glucoside), nine flavonols, seven phenolic acids (mainly caffeoylquinic acids), six flavan-3-ols, five flavones, and two flavanonols (dihydroflavonols). According to these authors, berries of different cultivars of honeysuckle also contained six simple iridoids such as loganic acid and its derivatives and nine secoiridoids, e.g., sweroside, loganin, and its derivatives. Additionally, Mech-Nowak et al. 2014 determined carotenoids such as lutein, zeaxanthin, and β-carotene in the berries of two cultivars of honeysuckle [25].

Bilberry fruits contain anthocyanins (derivatives of delphinidin, cyanidin, petunidin, peonidin, and malvidin), phenolic acids, and also carotenoids, mainly lutein.

### 3.1. The Effect of Using the Test Product (AKB) in the First and Second Stages of the Study

The first part of the analysis checked what the effect of using the study product was. For this purpose, a frequency analysis was performed for the results of supplementation, and then, the chi-square test of concordance was used to verify whether there were differences in the frequency of the positive effect in the study group. The results of the analysis are shown in Table 1.

The analysis showed a statistically significant positive effect advantage for near visual acuity in the second stage of the study, and a statistically significant (at the level of statistical trend) positive effect advantage for Schirmer test results in the first stage. 

**Figure 3:**
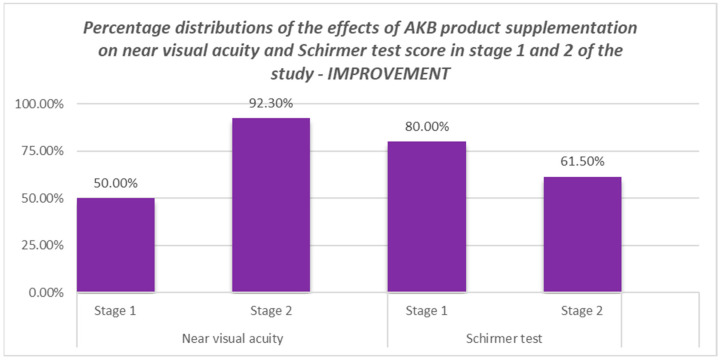
Comparison of the study group with the control group in terms of the occurrence of improvement in the Schirmer test and near vision acuity.

It also turned out that in terms of near visual acuity in the first stage and Schirmer test results in the second stage, the frequency of positive effect was similar to the frequency of non-positive effect. The results of the analysis are illustrated in Figure 3.

### 3.2. Comparison of the Study Group with the Control Group in Terms of the Occurrence of Improvement in Near Visual Acuity in the First and Second Stages of the Study

The test group (AKB test product) was then compared with the control group (placebo) in terms of the occurrence of improvement in near visual acuity. For this purpose, a chi-square test of independence was performed. The results are shown in Table 2.

**Figure 4:**
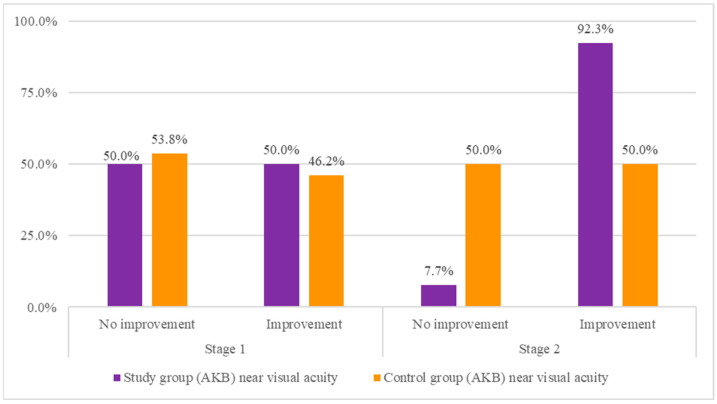
Comparison of the study group with the control group in terms of the occurrence of improvement in near vision acuity.

The analysis showed a statistically significant difference between the compared groups in the frequency of occurrence of improvement in near visual acuity at the second stage. It turned out that those in the study group significantly more often experienced a positive effect compared to those in the control group. Moreover, the recorded effect was found to be moderately strong (0.30 < ϕ < 0.50). In the case of stage one, there was no statistically significant difference between the compared groups, meaning that the positive effect occurred at a similar frequency in both the study and control groups. The results of the analysis are illustrated in Figure 4.

#### Percentage Distributions of Supplementation Effects on Near Visual Acuity in the Test and Control Groups

Next, the test group (test product) was compared with the control group (placebo) in terms of the occurrence of improvement on the Schirmer test. For this purpose, a chi-square test of independence was performed. The results are shown in Table 3.

**Figure 5:**
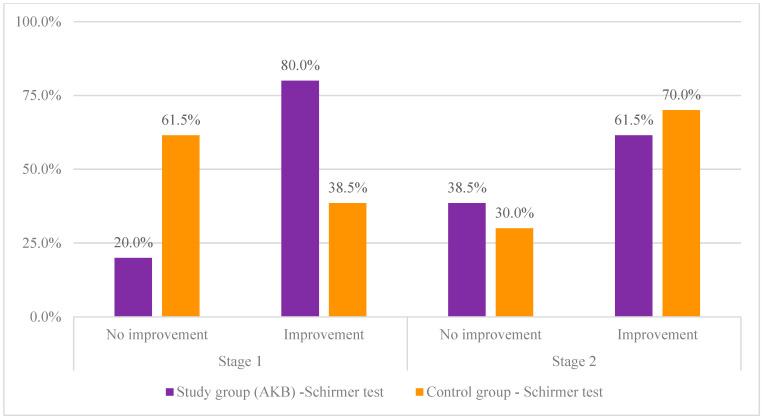
Percentage distributions of supplementation effects on Schirmer test scores in the test and control groups.

The analysis showed a statistically significant difference between the compared groups in the frequency of occurrence of improvement in the Schirmer test score at the first stage. It turned out that those in the test group significantly more often experienced a positive effect compared to those in the control group. Moreover, the recorded effect appeared to be moderately strong (0.30 < ϕ < 0.50). In the case of the second stage, there was no statistically significant difference between the compared groups, which means that in both the study group and the control group, the positive effect occurred with similar frequency. The results of the analysis are illustrated in Figure 5.

At this preliminary stage of the study, no differences were seen in the effect of the study preparation on visual acuity to distance, sense of contrast to distance and near, intraocular pressure, or visual field. A slight improvement was noted in the other tests assessing ocular lubrication: LIPCOF and tBUT.

## 4. Discussion

The fruits of chokeberry *Aronia melanocarpa* have widely described uses. They exhibit a wide range of bioactivities potentially beneficial to human health, including anti-diabetic, anti-infective, anti-cancer, anti-obesity, and antioxidant effects, as well as gastrointestinal, cardiac, liver, and neuroprotective effects [27,28,29,30]. To date, phenolic compounds such as anthocyanins, cyanidins, phenolic acids, proanthocyanidins, triterpenoids, and their analogs have been identified as the main active constituents of chokeberries [26,31]. Reports on the effects of chokeberry on the organ of vision are scarce. The possible mechanism mode of action of *A. melanocarpa* by which anthocyanins and proanthocyanines could accelerate biosynthesis of rhodopsin, modulate activity of retina enzymes, and improves microcirculation [32]. It is known that anthocyanin-containing chokeberry fruit protects the rat retina in a NaIO_3_-induced dry AMD model [33]. Chokeberry extract has a dose-dependent anti-inflammatory effect on the organ of vision [18]. An extract of the honeysuckle berry attenuates ocular inflammation in an animal model of endotoxin-induced uveitis by inhibiting the NF-kappaB-dependent signaling pathway and subsequent production of pro-inflammatory mediators [34]. The bilberry (blueberry, *Vaccinium myrtillus*) of the heather family (Ericaceae) has the most documented effect on the condition of the organ of vision. It is one of the richest natural sources of anthocyanins. The use of bilberry is most commonly described for improving vision. However, it is known to have other properties: it lowers blood glucose levels, has anti-inflammatory and lipid-lowering effects, promotes antioxidant defense, and reduces oxidative stress [10].

Bilberry has been widely used to improve night vision and reduce vascular permeability and capillary fragility; the greatest interest of researchers has focused on the antioxidant effects associated with anthocyanins [10]. A number of studies have shown positive effects, including improved retinal abnormalities, increased capillary resistance, slowed progression of lens opacity and myopia, and improved adaptation to darkness. For example, in a study involving 50 patients with mild senile cataracts, 4-month supplementation with bilberry anthocyanins and vitamin E showed 97% efficacy in preventing cataract progression [10]. Long-term consumption of bilberry extract can slow axial elongation and control myopia progression in children with high myopia [35]. Despite the many studies that have been conducted, as with many other substances of natural origin, large multi-center studies are lacking, and the active ingredients in blueberries do not have the same potent effects as chemical drugs. The published studies focus on healthy people without eye disease [36].

Nevertheless, there is scientific evidence supporting the beneficial effects of blueberries in relation to ocular disorders and vision loss. Bilberry anthocyanins (1 mg/mL) modulated oxidative stress defense enzymes heme oxygenase-1 (HO)-1 and glutathione-S-pi transferase (GST-pi) in retinal pigment epithelial cells that were pre-incubated with anthocyanin extract before H_2_O_2_ challenge. In a study with cultured retinal ganglion cells, bilberry anthocyanosides inhibited chemically induced cell damage and radical activation, and the observed neuroprotective effect, which may have been related to antioxidant activity, was also described in in vivo studies when bilberry anthocyanosides (100 μg/eye) were injected into the vitreous body of mice [10]. Bilberry anthocyanins have been shown to modulate the oxidative-stress-protective enzymes heme oxygenase-1 (HO-1) and glutathione S-transferase-pi in human retinal pigment epithelial cells [33].

Asthenopia and contrast sensitivity symptoms in 22 of 30 subjects (73%) improved significantly after 4 weeks of taking 100 mg/day of purified anthocyanin (85% anthocyanoside oligomers [10]. To date, the mainstay of presbyopia treatment is mainly ocular correction. However, in recent years, two types of pharmacological effects on presbyopia have been studied: pupil constrictors and lens softening agents (e.g., 1.5% lipoic acid choline ester) used in eye drops [3,5,8,37,38].

Pyrenoxine is used in eye drops in humans to slow the progression of cataracts. A significant inhibition of lens hardening was observed in rats administered a formulation of pyrenoxine in eye drops. Therefore, it has been suggested that the administration of pyrenoxine eye drops may prevent presbyopia [39]. Various dietary components have also been studied to slow presbyopia. Kono et al. investigated the effects of dietary supplements containing lutein, astaxanthin, cyanidin-3-glucoside, and docosahexaenoic acid (DHA) on improving the accommodative abilities of 48 people aged 45–64 who suffered from daily eye fatigue. The authors emphasized that a dietary supplement has an effect on improving accommodative abilities and subjective symptoms related to eye fatigue [40].

One of the few studies of natural compounds for inhibiting presbyopia involves the use of a herbal drug mixture containing *Cassiae Semen* (200 mg), wolfberry (200 mg), and *Dendrobium huoshanense* (40 mg) in a single capsule. The study included 400 participants between the ages of 45–70. The results of the study showed an increase in the amplitude of accommodation, demonstrating that the study preparation can delay or alleviate presbyopia. The authors proposed that the mechanisms for the improvement in near visual acuity may be due to effects on accommodations, rather than depth of acuity caused by miosis and the pinhole effect [41]. In the Chinese patent CN101450176A, a mixture of 15 Chinese herbs, including Radix Astragali, Rhizoma Alismatis, and Korean Ginseng, administered orally, can be used to treat presbyopia [42].

Effects on the parasympathetic nervous system are possible through two types of receptors: muscarinic and nicotinic. Natural and synthetic flavones and flavonols were assayed in vitro for their ability to inhibit radioligand binding at human cloned M1 muscarinic receptors. Several compounds were found to possess competitive binding affinity (Ki = 40–110 µM), comparable to that of acetylcholine (Ki = 59 µM) [43]. It was also observed that polyoxygenated flavones from the bark extract of *Melicope subunifoliolata* Hartley show competitive binding activity at muscarinic acetylcholine receptors [43,44]. A 2018 review study based on the results of peer-reviewed articles found that more than 70 extracts or components of medicinal plants affect muscarinic receptors (stimulate or inhibit) of smooth muscle in the cardiovascular, gastrointestinal, respiratory, and genitourinary systems [45]. Effects on the parasympathetic system can also be achieved by affecting the activity of the cholinergic system neurotransmitter acetylcholine. Khan et al. discuss a number of plant-derived flavonoids with exceptional efficacy and potency as AChE inhibitors and their safety profiles and pharmacokinetic properties [46]. The effect of flavonoids, including cyanidin-3-O-glucoside, is known to interact with M1/M2 muscarinic receptors in the CNS. These receptors are also found in the lacrimal glands of the eye. The effect of the extract may be related to this fact [47]. At the same time, some of the iridoids, such as loganin and sweroside (the dominant compound of this group in the extract), can effectively block cholinergic muscarinic receptor [48]. Interactions of dicaffeoylquinic acids with nicotinic receptors are described [49]. A paper by Brinz I et al. described similar effects of sweroside on *n*ACh receptors [50].

The lacrimal gland is the major contributor to the aqueous layer of the tear film which consists of water, electrolytes, and proteins. Small changes in the concentration of tear electrolytes have been correlated with dry eye syndrome. While the mechanisms of secretion of water, electrolytes, and proteins from the lacrimal gland differ, all three are under tight neural control. This allows for a rapid response to meet the needs of the cells of the ocular surface in response to environmental conditions. The neural response consists of the activation of the afferent sensory nerves in the cornea and conjunctiva to stimulate efferent parasympathetic and sympathetic nerves that innervate the lacrimal gland. The important receptors present on the lacrimal gland are acetylcholine receptors like muscarinic M3, vasoactive intestinal peptide types I and II, and norepinephrine receptors like alpha 1 and beta. Neurotransmitters are released from the stimulated parasympathetic and sympathetic nerves that cause secretion of water, electrolytes, and proteins from the lacrimal gland and onto the ocular surface [51]. 

The surgical removal of the lacrimal gland in mice and rats is the most commonly reported model in DED research [52]. 

Compounds with similar structures to those shown in Figure 2 have proven effects on the lacrimal gland. Thus, Inaba et al. proved the positive effect of another flavonoid—quercetin—and oral intake of quercetin-rich products on the lacrimal gland in studies on both mice and healthy volunteers [53]. In contrast, Ng et al. used standardized blueberry anthocyanins (4.5 mg/dose), finding a clinical reduction in dry eye disease (DED) symptoms (OSDI score) and improvement in objective tear film assessments such as NIF-BUT, NiAvg-BUT, lysamine green staining, and Schirmer test [54]. 

In Hitoe S et al., MaquiBright™ standardized maqui berry extract significantly increased tear fluid production and ameliorated dry eye-related symptoms in a clinical pilot trial [20]. Based on preclinical studies, the authors concluded that the etiology of dry eye syndrome includes oxidative stress exerted on the lacrimal gland, and antioxidants (here, the authors studied the effect of *Aristotelia chilensis* berry extract) help restore the activity of this gland. Moreover, it should be remembered that the tear film does not consist only of an aqueous component secreted by the lacrimal gland, but is a complex mixture that also contains a lipid component secreted by the Meibom’s glands, electrolytes, mucin, and proteins. The evaluation of tear film stability function is therefore not based on an isolated assessment of the activity of the gland itself.

The human eye is constantly subjected to oxidative stress due to daily exposure to sunlight, high metabolic activity, and oxygen pressure. The surface of the eye, consisting of the tear film, cornea, and aqueous fluid, forms the first physical and biochemical barrier of the eye and plays a key role in combating free radicals. These compartments of the eye are enriched in certain antioxidants in the form of metabolic enzymes or small molecules. Such an antioxidant defence system at the ocular surface is essential for maintaining redox homeostasis in the eye and protecting against oxidative damage [55]. Oxidative stress has been described as an important pathomechanism of many degenerative eye diseases [56]. Various oxidative damages (e.g., lipid peroxidation, DNA damage, autophagy, and apoptosis) have been described to occur in different areas of the eye (e.g., cornea, anterior chamber, lens, retina, and optic nerve). The antioxidant mechanisms present in the eye are known, as are the ocular diseases arising from antioxidant imbalance (e.g., corneal cone, cataract, age-related macular degeneration, and glaucoma), the relevant antioxidant biomarkers and the potential for predictive diagnostics. The literature also describes natural antioxidant therapies for oxidative stress-related eye diseases [56,57,58,59,60]. 

The mechanism of antioxidant compounds on presbyopia has also recently been demonstrated. Nakazawa et al. demonstrated that a compound structurally similar to those found in the preparation studied, the flavonoid α-glucosyl-hesperidin orally administrated prevents lens hardening [61]. On a rat model, it was proven that it reduces aging-induced oxidative stress in the lens and plasma and ameliorates cataract development by preventing sclerosis of the eye. It was confirmed that it upregulated the mRNA expression of anti-oxidative enzymes such as glutathione reductase and thioredoxin reductase 1 in the plasma and lens.

Preparations containing natural compounds are also being studied for use in eye fatigue conditions. One study evaluated the effects of a preparation containing esters of lutein, zeaxanthin, and extracts of black currant, chrysanthemum, and goji berries. A reduction in eye fatigue symptoms was observed, including eye pain, blurred vision, dry eyes, foreign body sensation, and tearing. An improvement in tear secretion confirmed by the Schirmer test was also noted in the study group compared to placebo [62]. Oral intake of bilberry extract for 8 weeks was shown to reduce eye fatigue caused by video display terminal (VDT) workload, including eye pain, eye heaviness, discomfort, and foreign body sensation, as reduced critical flicker fusion was alleviated after 8 weeks of bilberry use. A longer period of bilberry extract consumption (12 weeks) alleviated tonic ciliary muscle accommodation caused by VDT screens and near workload [35]. Bilberry extract can alleviate the symptoms of dry eye syndrome. With its supplementation, dry eye syndrome patients showed an increase in tear secretion volume over four weeks [35].

Dietary supplementation with bilberry extract and fish oil for 3 months showed improvement in signs and symptoms of severe dry eye, which was independent of age [63].

Natural compounds of animal origin are also being investigated for the treatment of dry eye syndrome. A natural extract consisting of bee venom, musk, and antler was effective in treating dry eye syndrome in rats by restoring the damaged eye surface, increasing tear volume, and rebuilding the mucin layer of the tear film [64].

An example of another plant that shows therapeutic activity in dry eye syndrome is an extract of *Bidens pilosa*, widely used in Chinese medicine. In the treatment of androgen-deficient dry eye in rats, it has been found to improve the amount of the aqueous part and maintain tear film stability, as well as inhibit inflammation of the lacrimal gland [65]. The role of diet (omega-3 fatty acids, flavonoids, carotenoids) on the state of the organ of vision is studied especially in users of VDT screen monitors [66]. Supplementation with bilberry extract improved some objective and subjective parameters of eye fatigue induced by screen monitor loads [67,68]. 

## 5. Conclusions

This is the first study of a preparation based on a combination of natural extracts of aronia, honeysuckle, and bilberry.

The results of the study showed that AKB used for 6 weeks did not change visual acuity during the first 6 weeks of treatment. It was not until the next cycle of the study that an improvement in near vision was observed in 92.3% of patients. This may indicate the potential of the formulation to correct near vision in patients with presbyopia. 

On the other hand, an improvement in conjunctival hydration in the Schirmer test was observed in 80% of patients as early as week 6 of treatment. This effect decreased to 61.5% in the following weeks.

The above observations are preliminary studies of AKB preparation, providing a premise for possible activity in the treatment of presbyopia as well as dry eye syndrome. Further in-depth studies on a suitable group of patients together with an understanding of the molecular mechanism of action may prove the activity of the investigational preparation as an effective dietary supplement.

## Figures and Tables

**Table 1:** Percentage distributions of the effects of AKB product supplementation on near visual acuity and Schirmer test score.

		N	%	χ^2^ (1)	*p*
Near visual acuity—Stage 1	Improve	5	50.0%	<0.01	1.000
	No improvement	5	50.0%		
Near visual acuity—Stage 2	Improve	12	92.3%	9.31	0.002
	No improvement	1	7.7%		
Schirmer test—Stage 1	Improve	8	80.0%	3.60	0.058 ^
	No improvement	2	20.0%		
Schirmer test—Stage 2	Improve	8	61.5%	0.69	0.405
	No improvement	5	38.5%		

^ result at the level of statistical trend (0.050 < *p* < 0.100).

**Table 2:** Comparison of the study group with the control group in terms of the occurrence of improvement in near visual acuity in the first and second stages of the study.

Near Visual Acuity		Study Group		Control Group					
		N	%	N	%	χ^2^	*p*	*df*	ϕ
Stage 1	No improvement	5	50.0%	7	53.8%	0.03	0.855	1	0.04
	Improve	5	50.0%	6	46.2%				
Stage 2	No improvement	1	7.7%	5	50.0%	5.25	0.022	1	0.48
	Improve	12	92.3%	5	50.0%				

N—number of observations; χ^2^—chi-square test result; *p*—statistical significance; *df*—degrees of freedom; ϕ—strength of effect index.

**Table 3:** Comparison of the study group with the control group in terms of the occurrence improvement on the Schirmer test in the first and second stages of the study of the study.

Schirmer Test		Study Group		Control Group					
		N	%	N	%	χ^2^	*p*	*df*	ϕ
Stage 1	No improvement	2	20.0%	8	61.5%	3.97	0.046	1	0.42
	Improve	8	80.0%	5	38.5%				
Stage 2	No improvement	5	38.5%	3	30.0%	0.18	0.673	1	0.09
	Improve	8	61.5%	7	70.0%				

N—number of observations; χ^2^—chi-square test result; *p*—statistical significance; *df*—degrees of freedom; ϕ—strength of effect index.

## Data Availability

The original contributions presented in the study are included in the article/supplementary material, further in-quiries can be directed to the corresponding author.

## Supplementary Materials

The following supporting information can be downloaded at: https://www.mdpi.com/article/10.3390/nu16070926/s1, Table S1: Anthocyanin, phenolic acid, iridoid, and flavonol content (mg/100 g fw) of the extract AKB by HPLC.
