# Supplementary material for: Extract from Aronia melanocarpa, Lonicera caerulea, and Vaccinium myrtillus Improves near Visual Acuity in People with Presbyopia
Source: Nutrients. 2024 Mar 23;16(7):926. doi: 10.3390/nu16070926 (PMC11013737; doi:10.3390/nu16070926)
Supplement: Supplementary file 1 [file nutrients-16-00926-s001.zip › nutrients-2864211-supplementary.pdf]

Table S1. Anthocyanin, phenolic acid, iridoid, and flavonol content (mg/100 g fw) of the extract AKB by HPLC.

No.	Compound	Content (mg/100 g)
**ANTHOCYANINS**		
1	Cyanidin 3,5-*O*-diglucoside	990.03
2	Delphinidin 3-*O*-galactoside	
3	Delphinidin 3-O-glucoside	741.62
4	Cyanidin 3-*O*-galactoside	12 609.56
5	Delphinidin 3-*O*-arabinoside	
6	Cyanidin 3-*O*-glucoside	9 424.60
7	Cyanidin 3-*O*-rutinoside	6 289.38
8	Cyanidin 3-*O*-arabinoside	
9	Peonidin 3-*O*-galactoside	182.00
10	Petunidin 3-*O*-arabinoside	
11	Pelargonidin 3-*O*-glucoside	548.96
12	Malvidin 3-*O*-galactoside	
13	Cyanidin 3-*O*-xyloside	627.19
14	Peonidin 3-*O*-arabinoside	409.01
15	Malvidin 3-*O*-glucoside	
16	Malvidin 3-*O*-arabinoside	182.93
Other anthocyanins		96.81
**Total anthocyanins**		**32 102.09**
**PHENOLIC ACIDS**		
1	3-Caffeoylquinic acid	1 939.94
2	3-*O*-*p*-coumaroylquinic acid	1 124.70
3	5-Caffeoylquinic acid	3 037.30
4	Coumaroyl iridoid glycosides	320.17
5	Dicaffeoylquinic acid	215.67
6	*p*-Coumaric acid derivative	26.74
Other phenolic acids		658.29
**Total phenolic acids**		**7 322.82**
**IRIDOIDS**		
1	loganic acid	1390.46
2	Loganic acid derivative	521.55
3	Loganin	2945.89
4	Sweroside	
5	Loganin derivative	671.51
**Total iridoids**		**5529.41**
**FLAVONOIDS**		
1	Quercetin-dihexoside 1	46.38
2	Quercetin-dihexoside 2	178.42
3	Quercetin 3-*O*-vicianoside	225.37
4	Quercetin 3-*O*-rutinoside	657.66
5	Quercetin 3-*O*-galctoside	812.65
6	Quercetin 3-*O*-glucoside	
7	Quercetin 3-*O*-glucuronide	243.05

8	Quercetin 3-*O*-robinobioside	
9	Quercetin 3-*O*-pentoside	
10	Isorhamnetin pentosylhexoside	13.75
11	Isorhamnetin 3-*O*-vicianoside	23.99
12	Isorhamnetin rhamnosylhexoside 1	26.13
13	Isorhamnetin rhamnosylhexoside 2	9.98
14	Myricetin	3.09
**Total flavonoids**		**2 240.48**
